# Acupuncture Treatment for Ipsilateral Hemichorea Associated With Non‐Ketotic Hyperglycemia: A Case Report

**DOI:** 10.1002/ccr3.72763

**Published:** 2026-05-24

**Authors:** Yeqing He, Huanqin Li, Qiufu Dai

**Affiliations:** ^1^ Graduate School, Beijing University of Chinese Medicine Beijing China; ^2^ Department of Traditional Chinese Medicine Beijing Tiantan Hospital, Capital Medical University, China National Clinical Research Center for Neurological Diseases Beijing China; ^3^ Department of Acupuncture and Moxibustion Beijing Hospital of Traditional Chinese Medicine, Capital Medical University, Beijing Key Laboratory of Acupuncture Neuromodulation Beijing China

**Keywords:** acupuncture, case report, hemichorea associated with non‐ketotic hyperglycemia, neurology

## Abstract

For hemichorea associated with non‐ketotic hyperglycemia, hypoglycemic therapy is the most commonly used and effective treatment modality. However, some patients may still present with residual chorea‐like symptoms after glycemic control. Alternative symptomatic treatments, such as dopamine receptor antagonists or antipsychotics, carry the potential to induce tardive dyskinesia, Parkinson‐like extrapyramidal symptoms, and other adverse reactions. Herein, we report a rare instance of ipsilateral hemichorea where the patient's symptoms demonstrated significant improvement following a combination of acupuncture and hypoglycemic therapy. A 78‐year‐old female was diagnosed with HC‐NH due to right limb hemichorea persisting over the past month. In conjunction with hypoglycemic treatment, acupuncture was administered. The frequency of right limb hemichorea notably decreased after 2 weeks. Acupuncture might serve as a potentially efficacious salvage treatment option for HC‐NH.

## Key Clinical Message

Hemichorea associated with non‐ketotic hyperglycemia (HC‐NH) is a rare extrapyramidal syndrome. Acupuncture might serve as a potentially efficacious salvage treatment option for HC‐NH.

**Abbreviations**
CT: computed tomography
DWI: diffusion‐weighted imaging
HC‐NH: hemichorea associated with non‐ketotic hyperglycemia
MRI: magnetic resonance imaging

## 1 Background

HC‐NH is an infrequently encountered extrapyramidal syndrome characterized by non‐ketotic blood glucose levels, lateral involuntary dance‐like movements, and the presence of a high signal intensity in the basal ganglia on one side of the brain in T1‐weighted MRI images or a high density on a CT scan [1]. It has been historically reported as more prevalent in elderly women, with a male‐to‐female ratio of 1:1.8, though a recent large case series suggests a more equal sex distribution [2].

Clinically, it is not commonly seen, and ipsilateral HC‐NH is even more rare. Treatment is often postponed due to misdiagnoses as epilepsy, hyperactivity disorders, or cerebrovascular diseases [3, 4]. The underlying pathogenesis of HC‐NH remains elusive. In the clinical management of HC‐NH, hypoglycemic therapy is the most widely utilized and effective approach. Nevertheless, some patients experience residual dance‐like symptoms after blood sugar regulation [5]. To address these residual manifestations, dopamine receptor antagonists (e.g., chlorpromazine) or antipsychotics (e.g., olanzapine) are frequently prescribed to ameliorate the involuntary dance‐like movements of the limbs. However, the utilization of such medications is associated with a variety of adverse effects [6]. Regarding the extrapyramidal system, common side effects include akathisia, acute dystonias, parkinsonism and tardive dyskinesia, affecting 10%–35% of patients [7]. In terms of endocrine side effects, hyperprolactinemia occurs in approximately 20%–40% of patients treated with antipsychotic medication [7]. Regarding metabolic effects, antipsychotic‐induced weight gain is often a neglected side effect in the treatment, which can increase the risk of developing metabolic syndrome, diabetes and cardiovascular disease [8]. Antipsychotics may have cognitive and mental effects, such as dysphoria and lower subjective well‐being, affecting domains including mental functioning, self‐control, emotional regulation, physical functioning, and social integration. Regarding cardiovascular risks, partial dopamine agonists can cause QTc prolongation. Additionally, postural hypotension occurs in up to approximately 25% of patients [7, 9].

Acupuncture has demonstrated effectiveness in treating nervous system disorders with minimal side effects, yet there are relatively few reports regarding its application in the treatment of HC‐NH. In this paper, a rare case of ipsilateral HC‐NH is presented to offer diagnostic and treatment insights for the acupuncture therapy of HC‐NH.

## 2 Case Report

The patient, a 78‐year‐old woman, was admitted to the acupuncture department of Beijing Hospital of Traditional Chinese Medicine on August 1, 2020, due to a one‐month history of involuntary, dance‐like movements of her right‐sided limbs. Higher cortical functions, including memory, calculation, understanding, and orientation, were impaired. Muscle strength assessment indicated normal limb muscle strength (rated 5). Bilateral Babinski and Chaddock signs were negative. The patient had a long‐standing history of type 2 diabetes mellitus but had not been taking any medications and reported no other relevant medical history. The admission random blood glucose was 18.6 mmol/L, and the glycosylated hemoglobin level was 16.4%. Urine ketone bodies were not found in the routine urine examination, and there was no evidence of acid–base imbalance in blood gas analysis. Therefore, diabetic ketoacidosis was excluded. A head CT scan conducted on August 2, revealed a high‐density shadow in the right caudate and lentiform nuclei, with a CT value of approximately 50HU. Metabolic lesions were initially suspected, and the possibility of cerebral hemorrhage could not be excluded. There was no evidence to support a diagnosis of Parkinson's syndrome or familial chorea disease. On August 5, the patient had a brain MRI, and the imaging exhibited patchy short T1 signals and long T2 signals within the right caudate nucleus and putamen nuclei. FLAIR images demonstrated hypointensity, and DWI also showed hypointensity in these regions, indicative of abnormal signal patterns consistent with metabolic lesions.

### 2.1 Treatment

**FIGURE 1:**
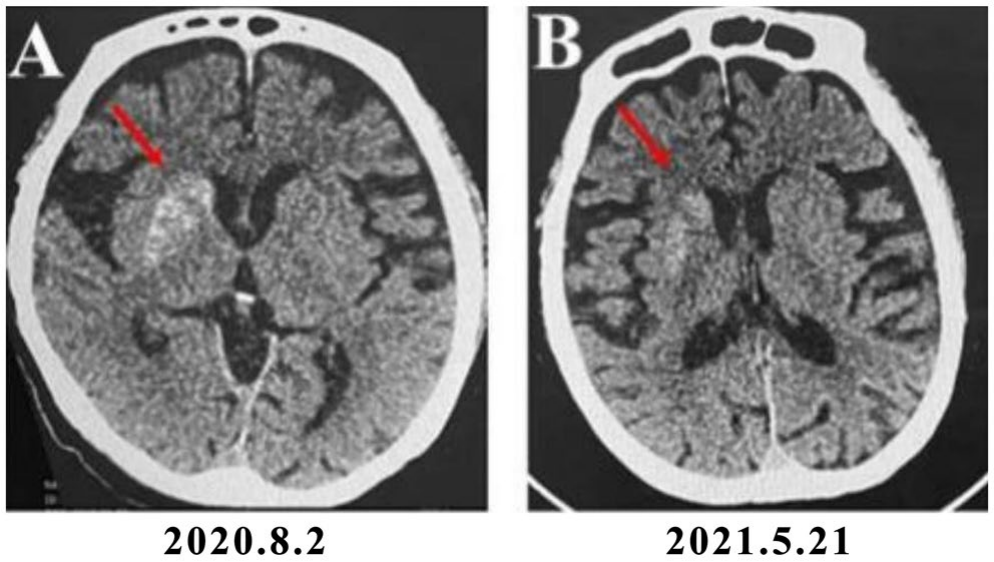
Serial non‐contrast axial head CT scans. (A) The initial scan from August 2, 2020, demonstrates a subtle hyperdensity in the region of the right caudate nucleus and putamen (red arrow), consistent with diabetic striatopathy. (B) A follow‐up scan performed on May 21, 2021, shows a marked reduction in the size and density of the lesion (red arrow), correlating with clinical improvement.

**FIGURE 2:**
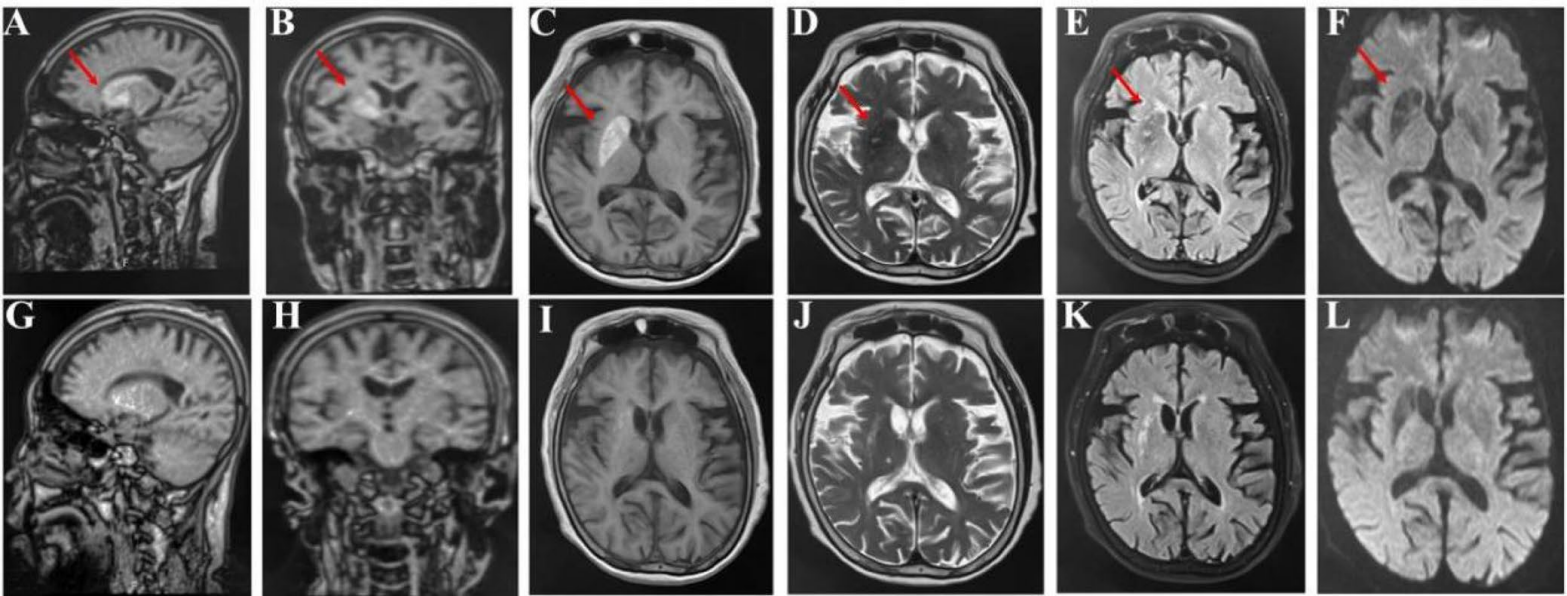
Serial brain MRI of the right basal ganglia. The top row (A–F) displays images from the initial presentation on August 5, 2020, while the bottom row (G–L) shows follow‐up imaging from May 25, 2021. The initial scan reveals a lesion in the right putamen (red arrows) characterized by distinct hyperintensity on T1‐weighted imaging (C) and corresponding hypointensity on T2‐weighted (D), T2‐FLAIR (E), and Diffusion‐Weighted Imaging (DWI) (F) sequences. The follow‐up imaging (G–L) demonstrates a near‐complete resolution of these signal abnormalities, particularly the T1 hyperintensity (I).

The patient received acupuncture treatment administered by a licensed acupuncturist on a daily basis. Traditional acupuncture treatment was based on the principles of sedation, regulating spirit, invigorating spleen, and assisting transportation. The acupuncture point prescriptions incorporated GV20, EX‐HN1, GV24, bilateral LI4, bilateral LR3, bilateral KI3, bilateral SP3, and bilateral ST36. Among them, GV20, EX‐HN1 and GV24 played a role in modulating cerebral function, while distal points, including LI4, LR3, KI3, SP3, and ST36, were chosen to address the underlying metabolic imbalance and constitutional deficiencies contributing to the manifestation of chorea. Upon needle insertion, stimulation techniques including lifting, thrusting, twirling, and rotating were applied to elicit the de qi sensation. When the patient experienced sensations of soreness, numbness, swelling, and pain, we considered that the de qi sensation had been achieved. The needles were retained in situ for 30 min. ANDE brand sterile acupuncture needles (40 mm in length and 0.3 mm in diameter) were utilized. Insulin aspartate injection (6–8–8 IU) was administered prior to each meal, along with Novolin N (12 IU) before bedtime. Two weeks after the initiation of acupuncture and blood glucose control, the frequency of right limb hemichorea had markedly declined. The patient's symptoms improved, and she was discharged. There was no recurrence of chorea after discharge. The patient was followed up for approximately 9 months. A second hospitalization occurred on May 20, 2021. A head CT scan performed on May 21, 2021, revealed an abnormal density in the right caudate nucleus. The lesion area was smaller, and the density was reduced compared to the images obtained on August 2, 2020 (Figure 1). A brain MRI conducted on May 25, 2021, demonstrated that the original abnormal signal in the right basal ganglia region was significantly diminished compared to the previous examination (Figure 2).

## 3 Discussion

The patient presented a typical case of HC‐NH with right limb hemichorea, which corresponds to the lesion site indicated by her imaging examination results, namely the right caudate nucleus of the brain. This case is highly unusual as, in most instances of HC‐NH, the lesions predominantly occur in the brain area on the opposite side of the affected limb. We believe this may be related to the structural characteristics of the extrapyramidal system. In the pyramidal decussation, 10%–20% of the fibers do not cross but continue their descent on the same side. Similarly, a portion of the descending fibers of the extrapyramidal system also follows an ipsilateral course [10]. The ipsilateral hemichorea observed in the patient may be associated with damage to the uncrossed corticospinal tract, together with disruption of the ipsilateral extrapyramidal projections and basal ganglia–thalamic circuits [11]. Su et al. [12] reported a similar case in 2012. HC‐NH is prone to misdiagnosis as cerebral hemorrhage during the initial treatment phase. Therefore, it is essential to conduct repeat head CT and MRI scans in a timely manner and combine them with clinical manifestations for accurate differentiation. With aggressive treatment, the prognosis for diabetic striatopathy is generally excellent [13]. A clinical retrospective study disclosed that among 12 HC‐NH patients whose lateral dancing symptoms subsided, 9 patients exhibited complete resolution of cranial imaging abnormalities after 11 months [14]. In this case, the patient with HC‐NH was treated with active hypoglycemic treatment, combined with traditional Chinese medicine acupuncture, without recourse to dopamine receptor antagonists and related sedative drugs. The involuntary dance‐like movements of the limbs were reduced. Repeat head CT and brain MRI examinations demonstrated a reduction in the extent of the lesions and an improvement in clinical symptoms, which align with the characteristic features of HC‐NH. Among them, blood sugar control is the core, clear and effective treatment measure, and acupuncture may be an effective adjunctive therapy to help improve symptoms. While it is impossible to disentangle the effects of the two concurrent therapies, this case suggests that acupuncture may represent a potential adjunctive therapy for managing the motor symptoms of HC‐NH, offering a potential approach for integrating traditional acupuncture into HC‐NH treatment. This approach may also help avoid the adverse effects associated with dopamine antagonists and antipsychotics.

## 4 Conclusion

Acupuncture therapy may serve as a beneficial adjunct therapy for HC‐NH. It may be considered as a complementary treatment to reduce the reliance on dopamine antagonists and antipsychotics, thereby avoiding their associated adverse effects. This observation warrants further investigation in larger, controlled studies to determine its potential efficacy and role in the treatment of this condition.

## Author Contributions

**Yeqing He:** writing – original draft. **Huanqin Li:** resources. **Qiufu Dai:** writing – review and editing.

## Funding

This work was supported by the Beijing Natural Science Foundation (grant no. 7252218) and the National Natural Science Foundation of China (grant no. 82205265).

## Ethics Statement

This study was conducted in accordance with the ethical principles of the Declaration of Helsinki. According to the policies of the Institutional Review Board of Beijing Hospital of Traditional Chinese Medicine, formal ethics approval is not required for a single retrospective case report.

## Consent

A written informed consent was obtained from the patient for publication of this case report and any accompanying images.

## Conflicts of Interest

The authors declare no conflicts of interest.

## Data Availability

The data that support the findings of this study are available from the corresponding author upon reasonable request.
